# Supplementary figures and images for: Bombyx mori Vps13d is a key gene affecting silk yield
Source: PLoS One. 2022 Jul 7;17(7):e0270840. doi: 10.1371/journal.pone.0270840 (PMC9262180; doi:10.1371/journal.pone.0270840)

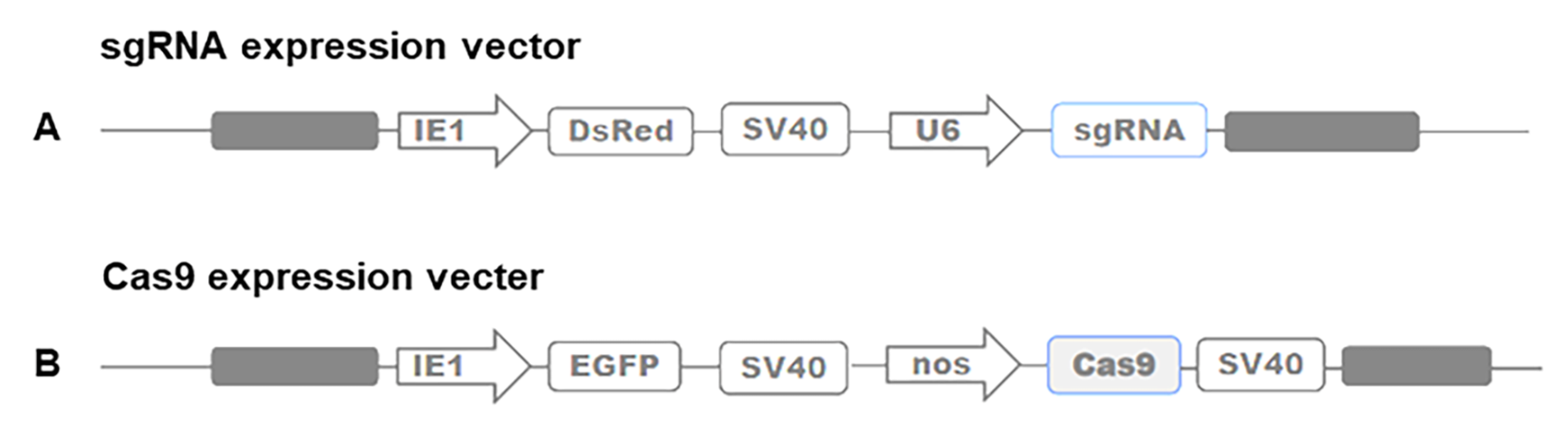

Supplement: S1 Fig — A, The sgRNA expression vector with red fluorescent protein (RFP) reporter gene; B, The Cas9 expression vector with enhanced green fluorescent protein (eGFP) reporter gene. (TIF) [file pone.0270840.s001.tif]

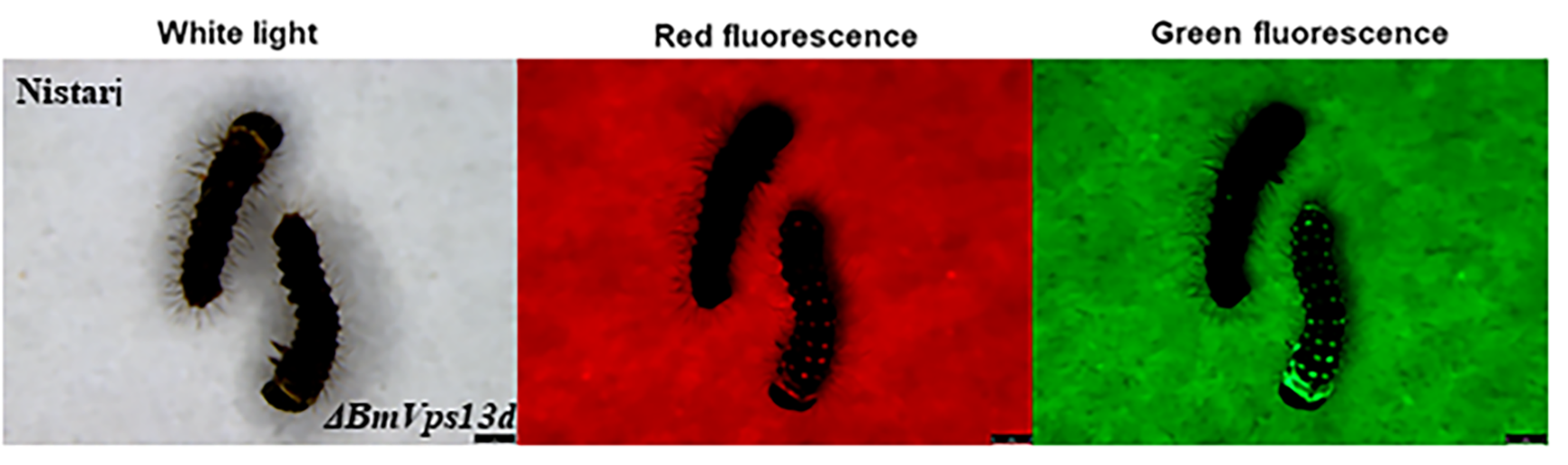

Supplement: S2 Fig — The picture shows the newly hatched first instar silkworm, scale bar = 1 mm. The red fluorescence, the ΔBmVps13d silkworm contains the sgRNA transgenic plasmid; the green fluorescence, the ΔBmVps13d silkworm contains the non-Cas9 transgenic plasmid. (TIF) [file pone.0270840.s002.tif]

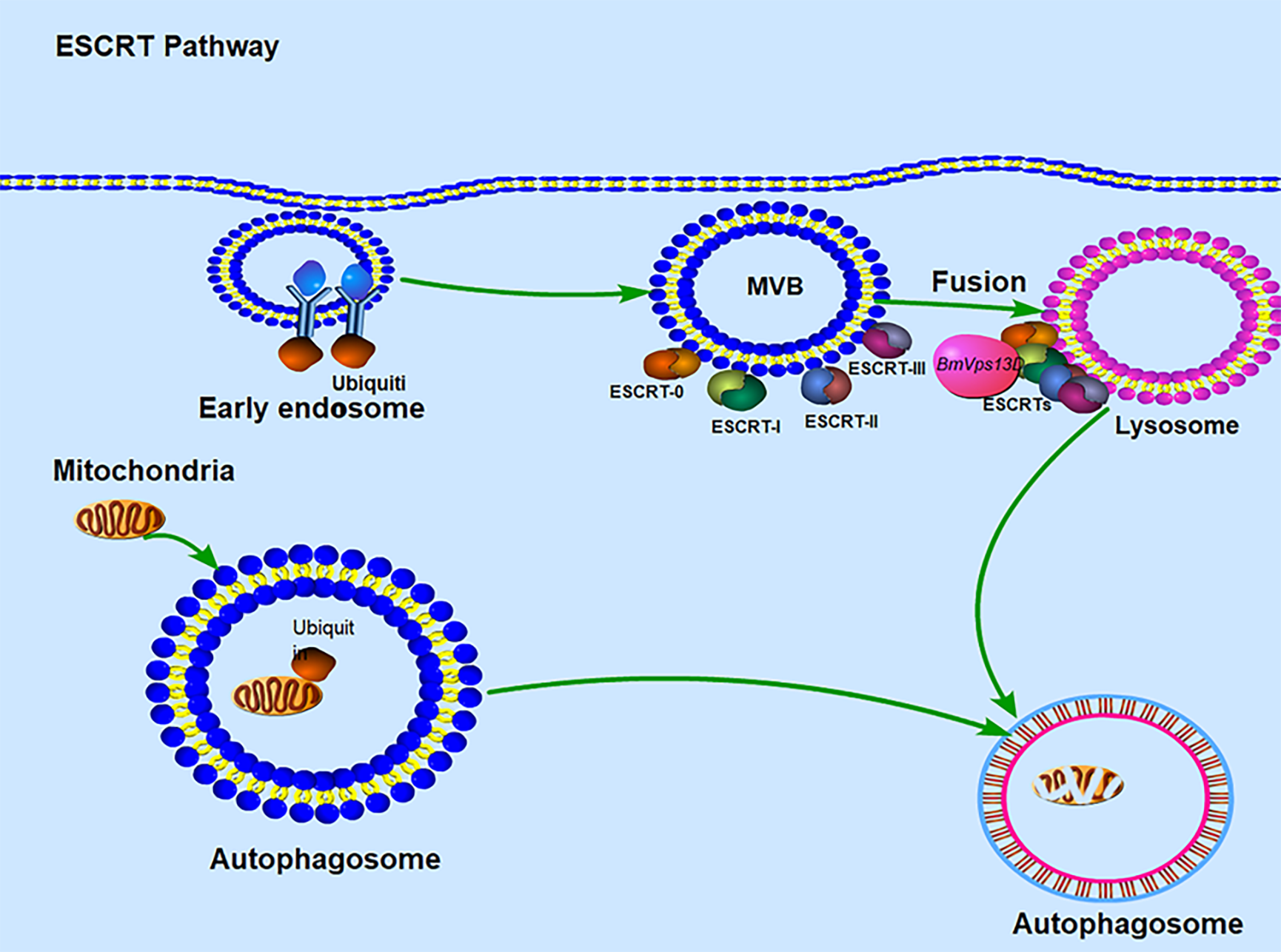

Supplement: S3 Fig — The picture shows the mechanism and process of mitochondrial autophagy and clearance mediated by BmVps13d in ESCRT pathway. (TIF) [file pone.0270840.s003.tif]
