# Supplementary material for: Bombyx mori Vps13d is a key gene affecting silk yield
Source: PLoS One. 2022 Jul 7;17(7):e0270840. doi: 10.1371/journal.pone.0270840 (PMC9262180; doi:10.1371/journal.pone.0270840)
Supplement: S1 Raw images — (PDF) [file pone.0270840.s005.pdf]

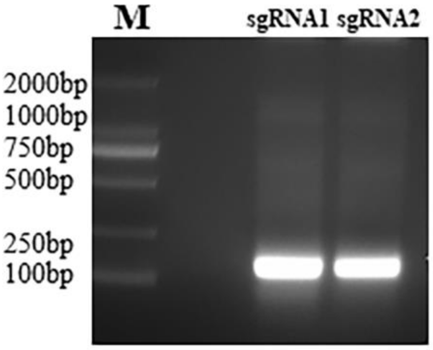

This original image corresponding to Fig. 5(B) in the main article.

PCR amplification of sequences containing the sgRNA-target sites (137 bp).

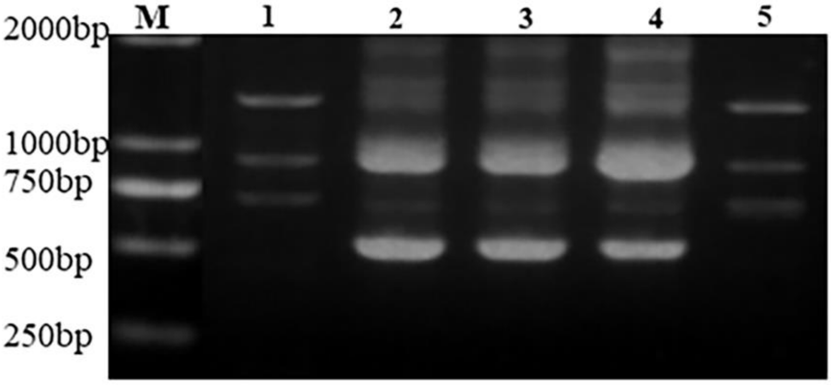

This original image corresponding to Fig. 5(C) in the main article.

Identification of the recombinant clones by PCR. Lane1, recombinant clone1 (1,337 bp) with sgRNA1 and sgRNA2; Lane5, recombinant clone2 (1,200 bp) with sgRNA1; Lane2-4, the failed recombinant clones.
