# Supplementary material for: Bombyx mori Vps13d is a key gene affecting silk yield
Source: PLoS One. 2022 Jul 7;17(7):e0270840. doi: 10.1371/journal.pone.0270840 (PMC9262180; doi:10.1371/journal.pone.0270840)
Supplement: Attachment — Submitted filename: Response to Reviewers.docx [file pone.0270840.s006.docx]

Reviewer #1: The silk yield is an important indicator to measure the quality of silkworm varieties. This paper determined that the difference in BmVps13d gene expression between JS and L10 may be related to the difference in promoter sequence, and verified that BmVps13d gene has an important effect on silk yield of silkworm using the CRISPR/Cas9 system. The findings are interesting and meaningful. It is acceptable after some revision.

1.Why were the sequence characterizations of Vps13d proteins compared in *B. mori*, *D. melanogaster*, and *H. sapiens* in Figure 1?

The functions of *Vps13d* have been reported in *D. melanogaster* and *H. sapiens*, this study predicted and compared the functional domain of this gene protein in *B. mori*, *D. melanogaster* and *H. sapiens.*

2. Line 178-180, “The results showed that the expression of BmVps13d were significantly higher in the midgut and silk gland of JS than in that of L10 at the third day of fifth instar larval stage (Fig 2)”. However, Figure 2 does not reflect the expression of BmVps13d significantly higher in the silk gland of JS than in that of L10. It is recommended to provide original data or change the plotting method.

The original data of Figure 2 has been provided in S4_Table.

3. Line 252-257, “The body weights of the ΔBmVps13d mutants showed a 24.7% decline than that of Nistari at the third day of fifth instar larval stage (Fig 7 A). ΔBmVps13d females showed 27.3%, 24.5%, and 24.8% declines in the weights of cocoons, pupae, and cocoon shells respectively; ΔBmVps13d males showed 11.9%, 4.3%, and 5.4% declines in the weights of cocoons, pupae, and cocoon shells respectively; 256 females and males of ΔBmVps13d showed 2.4% and 2.9% declines in the cocoon layer ratio respectively (Fig 7 B).” It is known that the body weight of the larvae directly affects the weight of cocoons, pupae, and cocoon shells. Knockout of BmVps13d resulted in reduced larval weight, which directly affected cocoons, pupae, and cocoon shells. Therefore, a direct relationship between the BmVps13d gene and cocoons, pupae, and cocoon shells cannot be demonstrated. Therefore, the conclusion needs to be revised.

The conclusion has been revised in lines 307-309.

4. It is recommended that authors analyze the expression of silk protein genes in Nistari and ΔBmVps13d mutants.

The expression of silk protein genes in Nistari and Δ*BmVps13d* mutants have been analyzed in

Lines 263-270.

Reviewer #2:

1. The promoter sequence in Figure 4 is the same as in Figure 3?

Yes，the promoter sequence in Figure 4 is the same as in Figure 3.
2. Page 6: lines 114-115: the role of pRL-TK vector in dual luciferase assay should be demonstrated.

The role of pRL-TK vector in dual luciferase assay has been demonstrated in lines 114.
3. Two sgRNA sites of BmVps13d were designed. Why was only one detected in Figure 6?

The target site2 missed the target and was not knocked out successfully.
4. Page 8: lines 158-160: Why were Δ*BmVps13d* mutants and Nistari weighed on the third day after cocooning?

During this period, silkworms have been completely cocooned, and phenotypic investigation is most appropriate at this time.

5. How about the effect of BmVps13d on the expression of silk protein genes? As an important information, the author should detect it.

The expression of silk protein genes in Nistari and Δ*BmVps13d* mutants have been detected in lines 263-270.
6. There are some writing errors in the manuscript, please proofread the full text carefully.

We proofread the full text and correct some writing errors in lines 11、14、20、28、70、79、82-83、165、167-170、181、232、292、298、308、309、311.
